# Minimally Invasive Ablation Strategies for Renal Cell Carcinoma Patients Ineligible for Surgery

**DOI:** 10.3390/life16010073

**Published:** 2026-01-04

**Authors:** Or Schubert, Maria Chiara Sighinolfi, Filippo Gavi, Enrico Panio, Simone Assumma, Antonio Silvestri, Giuseppe Pallotta, Vincenzo Cavarra, Pierluigi Russo, Nazario Foschi, Eros Scarciglia, Alessandro Posa, Alessandro Maresca, Gaetano Gulino, Alessandro Cina, Chiara Ciccarese, Roberto Iacovelli, Roberto Iezzi, Bernardo Rocco

**Affiliations:** 1 A. Gemelli University Hospital Foundation IRCCS, 00168 Rome, Italy; 2 Department of Urology, Fondazione Policlinico Universitario Agostino Gemelli IRCCS, 00168 Rome, Italy; 3 Department of Life Science, Health and Health Professions, Unilink University, 00165 Rome, Italy; 4 Department of Diagnostic Imaging and Radiation Oncology, Fondazione Policlinico Universitario Agostino Gemelli IRCCS, 00168 Rome, Italy; 5 Department of Oncology, Fondazione Policlinico Universitario Agostino Gemelli IRCCS, 00168 Rome, Italy

**Keywords:** renal cell carcinoma, radiofrequency ablation, microwave ablation, cryoablation, high-intensity focused ultrasound, minimally invasive therapies, percutaneous techniques

## Abstract

Minimally invasive ablative therapies have emerged as effective and safe alternate approach for the management of renal cell carcinoma (RCC), particularly in patients who are ineligible for surgery due to comorbidities or high operative risk. Techniques such as radiofrequency ablation (RFA), microwave ablation (MWA), cryoablation (CA), and high-intensity focused ultrasound (HIFU) offer kidney-sparing treatment with reduced morbidity. Current evidence suggests that for cT1a tumors (<4 cm), thermal ablation achieves technical success rates exceeding 95%, with local recurrence rates ranging from 1% to 9% and major complication rates generally below 5–7%. RFA is particularly suitable for small peripheral tumors, MWA enables rapid and deeper heating for larger or more vascular lesions, and CA provides precise control near critical structures. HIFU remains largely experimental with limited clinical applicability. Overall, these strategies demonstrate favorable oncological outcomes, emphasizing the importance of careful patient selection, multidisciplinary evaluation, and further studies to refine technique-specific indications and integration with systemic therapies.

## 1. Introduction

RCC accounts for about 90% of kidney cancers and is more common in men, typically diagnosed in older adults. Major risk factors include smoking, obesity, hypertension, and metabolic syndrome [1,2,3]. The main histological subtypes are clear cell, papillary, and chromophobe RCC, with 5-year survival rates of approximately 81%, 82%, and 91%, respectively [4,5]. Over half of RCC cases are incidentally detected during imaging for unrelated conditions, while the classic triad of flank pain, mass, and hematuria occurs in less than 10% of patients [6].

Surgical resection, either partial or radical nephrectomy, remains the gold standard for localized disease. However, minimally invasive alternatives such as cryoablation and radiofrequency or microwave ablation are increasingly used in patients with small tumors (<4 cm) or high surgical risk. Compared with nephrectomy, these thermal ablation techniques offer reduced morbidity, faster recovery, and better renal function preservation [7,8].

This review summarizes current evidence on indications, techniques, complications, and oncologic outcomes of thermal ablation in RCC. While systematic reviews and meta-analyses provide statistical synthesis, they often lack the flexibility to integrate technical nuances with complex clinical decision-making. The rationale for selecting a narrative format was to address this gap, offering a comprehensive, head-to-head comparison of all available ablative modalities (RFA, MWA, CA, HIFU) specifically tailored for urologists managing high-risk candidates. Furthermore, while partial nephrectomy remains the gold standard, it is associated with perioperative complication rates of up to 20% and potential renal function decline [7,9]. In frail, elderly, or solitary-kidney patients, these surgical risks often outweigh the oncological benefits, necessitating a shift towards less invasive, nephron-sparing strategies that prioritize quality of life and organ preservation.

## 2. Methods

For this narrative review, a comprehensive literature search was conducted using the PubMed, Scopus, and Cochrane Library databases. The search was restricted to relevant articles published in English between January 2020 and April 2025. Key search terms included: “Renal Cell Carcinoma”, “Radiofrequency ablation”, “Microwave ablation”, “Cryoablation”, “High-Intensity Focused Ultrasound”, “Minimally invasive therapies”, “percutaneous techniques”.

Inclusion Criteria: We prioritized randomized controlled trials, prospective observational studies, and recent meta-analyses comparing ablative techniques to partial nephrectomy. Retrospective series were included only if they involved large cohorts (>100 patients) or long-term follow-up (>5 years); however, preliminary data from smaller studies were included for emerging technologies such as HIFU. Case reports and conference abstracts were excluded.

## 3. Indications and Ablative Approaches

Patient selection is a crucial pre-requisite to guarantee an effective ablation. It requires a robust multidisciplinary team approach involving urology, radiology, and oncology. This collaborative assessment allows for a comprehensive evaluation of oncological risk, technical feasibility, and surveillance strategies, ensuring that treatment decisions are not solely based on tumor size but also on anatomical complexity and patient frailty [10]. Some laboratory tests should be run before the procedure, such as platelet count (should be >50,000/µL), INR ratio (should be <1.5), creatinine, and estimated glomerular filtration rate. Since the treatment is generally conducted under sedation, a physical and pharmacological history should be acquired in order to check allergies or interactions with other medications [10].

The Latest European Association of Urology (EAU) guidelines recommend renal mass ablation as a suitable option for patients with RCC measuring less than 4 cm in diameter (cT1a), whether exophytic or endophytic, who are either ineligible for or decline radical nephrectomy or general anesthesia [9,10]. Ideal candidates often include individuals with significant comorbidities, advanced age, or a solitary kidney [11]. Additionally, patients with pre-existing renal impairment, multiple health conditions, or hereditary RCC syndromes such as Von Hippel-Lindau disease may also benefit from thermal ablation [8]. Renal mass ablation can also be considered in patients who have previously undergone partial nephrectomy or tumor enucleation, as well as in cases of recurrent lesions within the same kidney following prior ablation [10] (Table 1).

However, patient selection implies specific exclusions. Ablative therapies are not recommended for patients with uncorrected coagulopathy or active infection [12]. Regarding tumor size, while EAU guidelines support ablation for cT1a tumors (<4 cm), technique selection is often size-dependent: RFA efficacy tends to diminish for tumors > 3 cm due to the heat-sink effect and smaller ablation zones, whereas MWA is often preferred for larger lesions (3–4 cm) due to its ability to generate higher temperatures and larger ablation volumes [9,13]. Consequently, tumors larger than 4 cm (cT1b) represent a relative contraindication, carrying higher recurrence rates, although they may be treated in selected cases using MWA or CA with multiple probes [12]. Lesions located in challenging anatomical regions, such as the renal hilum or near the proximal ureter, also pose higher risks of complications.

Before proceeding with ablation, confirming the malignancy of the lesion is essential. This can be achieved through a pre-procedural biopsy, which may need to be repeated if initial results are inconclusive, or through imaging-based assessments. Standard radiological criteria, including contrast enhancement and a growth rate exceeding 0.8 cm per year during active surveillance, are key indicators of potentially aggressive tumor behavior [10]. However, renal tumor biopsy is not devoid of limitations. According to EAU guidelines, the non-diagnostic rate ranges from 10% to 22% depending on center experience [9]. Furthermore, there is a potential risk of sampling error, particularly in heterogeneous tumors where high-grade components might be missed. Despite these challenges, the concordance between biopsy and final pathology for malignancy is high (>90%), making it a mandatory step before ablation to avoid treating benign lesions [9,10].

The three primary percutaneous thermal ablation techniques are RFA, MWA and CA. Additionally, emerging technologies such as HIFU are transitioning from experimental research to clinical application. These procedures can be performed under either general anesthesia or conscious sedation. The primary goal of thermal ablation is to achieve coagulative necrosis, effectively destroying tumor tissue while minimizing damage to surrounding healthy structures [9,10,11,12].

## 4. Radiofrequency Ablation

RFA has been established as a minimally invasive technique for solid tumor treatment since the early 1990s and is now widely used in oncology [14].

The technique involves inserting a needle electrode into the tumor under ultrasound or CT guidance. A high-frequency alternating current (400–460 kHz) induces ionic oscillation, heating tissue to 50–90 °C. Temperatures above 100 °C are avoided to prevent vaporization and charring, which reduce efficacy [11,12,13,14].

The electrode is kept at target temperature for several minutes, destroying tumor cells and cauterizing surrounding vessels, useful in highly vascular tumors. The thermal field extends slightly beyond the tumor margins, ensuring complete ablation [11].

A major limitation is the heat-sink effect, where blood flow near large vessels (≥3 mm) disperses heat, lowering efficacy. However, this can protect nearby critical structures, especially in perihilar tumors. RFA’s coagulative properties also minimize bleeding risk. To protect the renal collecting system, a double-J catheter may be placed to circulate cold water during ablation [10,11,12,14].

Consequently, the efficacy of RFA is physically constrained by tissue impedance and local blood flow. Due to the heat-sink vulnerability, RFA is currently considered the reference standard primarily for small (<3 cm), peripheral, and non-vascular lesions, while its utility diminishes for larger or highly vascularized masses [10,11,12].

## 5. Microwave Ablation

MWA utilizes specialized coaxial or helical antennas inserted into the tumor under ultrasound or CT guidance. These emit electromagnetic waves (915 MHz or 2.45 GHz), causing rapid water molecule oscillation and frictional heating. Temperatures above 60–100 °C induce coagulative necrosis, protein denaturation, and tumor cell death [10,12].

Selection criteria resemble those for RFA, but MWA is often preferred for larger tumors (typically 3–4 cm) because it achieves higher temperatures faster and is less influenced by the heat-sink effect. It is especially effective for highly vascular or perivascular lesions [10,12,13].

MWA offers rapid, active heating that is independent of tissue conductivity, making it effective for cystic or necrotic lesions. However, real-time visualization is more limited compared to cryoablation. Furthermore, the high-energy delivery requires caution; the ablation zone propagates quickly and can be less predictable in shape, necessitating precise probe placement to avoid unintended thermal injury to adjacent structures [14,15,16].

## 6. Cryoablation

Unlike other ablation methods, CA is the only non-thermal approach to tumor destruction. The procedure is typically performed under general anesthesia, conscious sedation, or even local anesthesia, as the cooling effect itself provides a degree of analgesia. Imaging guidance, usually via CT or ultrasound, is essential for precise probe placement and monitoring [10,12].

Modern cryoablation primarily relies on the circulation of cryogenic fluids such as argon or nitrogen through specialized probes. These gases rapidly expand, reaching temperatures as low as −190 °C, a process governed by the Joule-Thomson effect. This extreme cold induces tumor cell death through multiple mechanisms, including direct physical damage to the cellular membrane from ice formation, initiation of stress response pathways, apoptosis and necrosis, vascular stasis during the thawing phase, and potential activation of immune responses [17,18]. Indications for cryoablation include small, localized tumors measuring < 4 cm, multiple tumors within a solitary kidney, tumor recurrence after prior partial resection or other ablation and tumors near critical structures (ex. renal hilum) due to real-time visualization of ice ball [10,12,17]. A key consideration, however, is that freezing does not induce immediate coagulation; thus, the risk of post procedural hematoma is theoretically higher compared to heat-based therapies, necessitating careful monitoring [17].

## 7. Mechanisms of Cell Death in Cryoablation

Tumor cell destruction during cryoablation results from a sequence of interconnected biological mechanisms, including direct structural injury from ice crystal formation, microcirculatory failure upon thawing, and activation of apoptotic and necrotic pathways [17]. Cells adjacent to the cryoprobe undergo rapid freezing, whereas peripheral cells experience slower cooling, resulting in the formation of both extracellular and intracellular ice crystals. Extracellular ice develops across varying freezing rates both near the cryoprobe and in peripheral regions. As extracellular fluid freezes, osmotic pressure rises, drawing water from the cytoplasm and causing cellular dehydration, metabolic disturbance, and membrane injury due to hypertonic stress [9,17].

During thawing, local hypotonicity triggers rapid water influx into dehydrated cells, leading to swelling and rupture. The consequent release of intracellular antigens and damage-associated molecular patterns (DAMPs) may elicit an immune response that enhances antitumor activity [17]. Intracellular ice forms mainly in regions exposed to the most rapid cooling, where insufficient time for dehydration allows ice crystallization within the cytoplasm. Upon thawing, small intracellular ice crystals coalesce into larger, unstable structures that intensify mechanical damage to membranes and organelles, promoting further cellular destruction [17].

In the peripheral ablation zone, repeated freeze–thaw cycles impair mitochondrial function, increasing pro-apoptotic proteins such as Bax and reducing anti-apoptotic BCL-2, thereby amplifying programmed cell death [17]. Finally, vascular stasis contributes significantly to tumor necrosis, post-thaw cessation of blood flow induces ischemia and further enhances cell death [17].

## 8. High-Intensity Focused Ultrasound (HIFU)

HIFU is a noninvasive therapeutic technique that has been studied for over 85 years. Unlike diagnostic ultrasound, which operates at lower intensities, HIFU delivers focused ultrasound waves at frequencies of 300 kHz to several MHz and intensities exceeding 1500 W/cm^2^, allowing for precise tissue ablation [19,20]. Despite the growing interest in HIFU as a noninvasive treatment for solid tumors, research on its application in renal tissue remains limited. While experimental studies have demonstrated successful ablation of both benign and malignant renal lesions, early clinical experiences encountered technical challenges, including skin burns and inconsistent tissue ablation outcomes [19,20].

## 9. Discussion

### 9.1. Clinical Context and Indications for Ablation

Surgical resection, either through radical or partial nephrectomy, remains the gold standard for the treatment of localized RCC. However, a substantial subset of patients is considered unsuitable for surgery due to advanced age, significant comorbidities, the presence of a solitary kidney, or high anesthetic risk particularly when tumors measure less than 3–4 cm. In these cases, minimally invasive thermal ablation techniques have emerged as viable alternatives, providing effective local tumor control while preserving renal function [8,9]. It is worth noting that international guidelines present diverging views on patient eligibility. While the EAU primarily restricts thermal ablation to elderly and comorbid patients who are unfit for surgery [9], the American Urological Association (AUA) offers a broader perspective. The AUA guidelines state that clinicians should consider thermal ablation as an alternate approach for all solid renal masses < 3 cm, regardless of surgical fitness, provided the patient is counseled on the potential for higher local recurrence. This highlights a shift in the U.S. towards prioritizing patient preference and reduced morbidity even in healthy candidates, whereas European practice remains more conservative [21].

Nevertheless, for the specific population analyzed in this review, patients ineligible for surgery, both guidelines strongly support the use of percutaneous ablation techniques (RFA, MWA, CA) due to their favorable safety profile and comparable oncologic outcomes in selected cases [22].

### 9.2. Complications and Postprocedural Considerations

After thermal ablation, early postprocedural imaging typically with computed tomography (CT) or ultrasound is essential to detect potential complications. Both heat-based and cold-based techniques demonstrate an increased risk of adverse events in larger lesions and centrally located tumors. Fortunately, most complications are minor and self-limiting, rarely requiring surgical or radiological intervention [8,9].

Hemorrhage is the most common complication, occurring in 1% to 18% of cases, but it is usually self-limited. Other possible complications include skin burns, infection, urothelial strictures, urine leakage particularly in endophytic or perihilar tumors and pneumothorax, which is more likely when targeting upper-pole renal masses near the diaphragm.

Tumors located near the bowel pose an additional risk due to potential thermal injury to adjacent organs. In such scenarios, the use of hydro dissection, a technique that involves injecting a protective fluid barrier between the tumor and surrounding tissues, has been shown to mitigate this risk by increasing spatial separation [11,12,23].

### 9.3. Efficacy and Comparative Outcomes of Ablation Techniques

Growing evidence supports the use of thermal ablation as a valid alternative to surgery for small renal masses, particularly in patients who are unfit for partial nephrectomy. Several recent studies have confirmed that percutaneous ablation techniques such as RFA, CA, and MWA offer oncologic outcomes comparable to surgery, with additional benefits including reduced morbidity, shorter hospital stays, and better preservation of renal function (eGFR, creatinine) [24,25,26].

Regarding oncological control, extensive retrospective multi-center studies and meta-analyses report local recurrence rates ranging from 1% to 9%, with outcomes heavily dependent on tumor size and operator volume [27,28,29]. Five-year cancer-specific survival ranges from 95% to 98%, comparable to partial or radical nephrectomy [9]. In contrast, for cT1b tumors (>4 cm), percutaneous ablation is associated with higher rates of local recurrence and major adverse events, emphasizing the importance of careful patient selection [9].

Recurrence rates vary by procedure: CA presents a local recurrence rate of approximately 7.2%, comparable to RFA in small masses. However, for RFA, recurrence risk is strongly size-dependent, rising from 4.2% in cT1a tumors to as high as 14.3% in cT1b cases, indicating that tumor stage plays a significant role. MWA has shown lower recurrence rates within the first year (typically 2–5%), likely due to its ability to create larger ablation zones, though rates tend to equalize by five years post-treatment [28].

Head-to-head comparisons between techniques are primarily based on retrospective series rather than randomized controlled trials. For cT1a tumors (<4 cm), RFA, MWA, and CA show comparable outcomes regarding local recurrence-free survival [9,10]. However, technique-specific advantages emerge in challenging scenarios: comparative data suggest that MWA may offer superior local control for larger tumors (>3 cm) compared to RFA, likely due to its resistance to the heat-sink effect [10,13]. Conversely, cryoablation demonstrates a lower risk of collecting system injury for central tumors [12,17]. Notably, CA is well tolerated and shows high primary efficacy rates (up to 98%) with low cancer-specific mortality, particularly for tumors between 3 and 4 cm [30,31,32,33,34].

Among ablation modalities, MWA has emerged as a particularly efficient option. Recent analyses show that MWA allows shorter procedural times compared to RFA and CA, with favorable oncologic results and a local tumor progression rate between 2% and 7% in T1a tumors [29,30]. Additionally, MWA demonstrated superior 10-year overall survival compared to RFA in long-term follow-up [27,29,30]. RFA remains a widely used modality with excellent outcomes in tumors smaller than 3 cm, showing technical success near 100% and minimal renal function loss [9,10,11]. While RFA may have slightly higher local recurrence than MWA in larger masses, it still provides strong cancer control in well-selected patients [35,36].

Understanding the factors associated with local recurrence is crucial for patient selection. Tumor size remains the most consistent predictor; recurrence rates significantly increase for tumors > 3 cm [36]. Beyond size, tumor location is critical: perihilar and central tumors pose a higher risk of incomplete ablation due to the “heat-sink” effect in thermal techniques (RFA, MWA), where adjacent blood flow dissipates energy. Histological subtype is another potential predictor, with some evidence suggesting that clear cell RCC may be more sensitive to thermal injury compared to non-clear cell subtypes, although data remains conflicting. Finally, operator experience plays a vital role; high-volume centers report significantly lower complication and recurrence rates, emphasizing the learning curve associated with these procedures [9,36].

Regarding HIFU, efficacy in native kidneys is limited by perinephric fat which absorbs ultrasound energy. A key study by the Oxford team on 17 patients (mean tumor size 2.5 cm) reported only a 30% volume reduction at 36 months, highlighting these limitations [19,20]. However, results appear superior in renal transplant recipients where perinephric fat is absent; notably, a case series demonstrated a 90% tumor volume reduction in a transplant patient after two sessions, suggesting a potential niche indication for this specific cohort despite the technique’s generally experimental status [19].

The majority of recurrences are observed within the first five years after ablation. Given that recurrence is generally more frequent after ablation than after surgical options, it is essential to implement structured follow-up protocols and ensure access to salvage treatments when necessary [28] (Table 2).

### 9.4. Cost-Effectiveness Analysis

Economic considerations are increasingly relevant in treatment selection. A recent cost-effectiveness analysis compared percutaneous cryoablation (PCA) against partial nephrectomy (PN) for cT1a tumors. The study found that PCA was significantly less costly, with overall expected costs of approximately $20,491 for ablation versus $26,478 for partial nephrectomy [37,38]. The primary drivers of this cost reduction are the shorter hospital stay and reduced operating room time. Furthermore, cryoablation remained the cost-effective strategy in over 84% of simulations, provided that local recurrence rates remained comparable [37,38].

### 9.5. Emerging Technologies: Combination of Ablative Techniques with Immunoablation

Another area that requires further investigation is the combination of immunotherapy with ablation techniques, which has shown promising potential, particularly in late-stage disease. Preliminary studies suggest that this approach may enhance the immune response against tumors. For example, a recent pilot study by Campbell et al. investigated the effects of tremelimumab with and without adjuvant cryoablation in 29 patients with metastatic RCC. The study demonstrated a significant increase in post-ablation immune cell infiltration, as well as an increased ratio of T effector cells to T regulatory cells within the tumor microenvironment [39].

### 9.6. Management of Recurrence and Salvage Options

Management options following ablation failure generally include repeat ablation, salvage surgery, or active surveillance, depending on the patient’s risk profile and tumor biology. According to the 2025 EAU Guidelines, salvage partial nephrectomy remains the preferred treatment when technically feasible, particularly if a patient’s clinical condition allows for surgical intervention [9]. Repeat ablation may be considered to offer disease control in selected cases where surgery remains too risky, whereas active surveillance is reserved for patients with slow-growing, asymptomatic recurrences who are poor candidates for further intervention.

Surgery in the post-ablation setting is technically challenging due to the inflammatory response caused by the primary treatment. The presence of perinephric fibrosis and dense tissue adhesions can make dissection difficult and may occasionally necessitate conversion to radical nephrectomy to ensure complete tumor removal [40].

Despite these anatomical difficulties, minimally invasive salvage procedures, such as laparoscopic or robotic partial nephrectomy, have shown high rates of local tumor control with acceptable complication profiles when performed in experienced centers [40]. Ultimately, while thermal ablation carries a slightly higher risk of initial recurrence compared to surgery, timely salvage treatment can maintain long-term oncologic outcomes and preserve renal function effectively [28,40].

## 10. Conclusions

Minimally invasive thermal ablation techniques have become a valuable alternative to surgery for RCC, particularly in patients with significant comorbidities or those at high surgical risk. RFA, MWA and CA provide effective local tumor control while preserving renal function, with oncologic outcomes comparable to partial nephrectomy in appropriately selected patients.

- RFA remains the reference standard for small (<3 cm), peripheral tumors due to its proven safety profile and extensive long-term data.
- MWA is increasingly preferred for larger (3–4 cm) or cystic lesions due to its higher power and resistance to the heat-sink effect.
- CA is the optimal choice for central or hilar tumors requiring precise margin visualization to spare the collecting system. Additionally, CA is the only technique indicated for the simultaneous treatment of multiple lesions.
- HIFU remains experimental and is not currently recommended for routine practice outside of clinical trials or specific cases like renal transplant recipients.

As these technologies continue to mature, the focus must remain on a personalized and multidisciplinary approach to ensure the optimal balance between oncologic control and functional preservation for every patient.

## Figures and Tables

**Table 1:** Patient and tumor selection criteria for thermal ablation.

Category	Criteria for Ablation Suitability
Patient factors	Elderly patients with limited life expectancy. High surgical risk due to severe comorbidities. Refusal of surgery. Genetic predisposition to multiple tumors (e.g., Von Hippel-Lindau disease). Solitary kidney or pre-existing chronic kidney disease (CKD).
Tumor factors	Small renal masses (cT1a, <4 cm). Recurrent tumors after partial nephrectomy. Favorable anatomy (distance from ureter/bowel).
Contraindications	Uncorrected coagulopathy. Active infection. Life expectancy < 1 year. Tumors > 4–5 cm (relative contraindication).

Note: These criteria are designed to guide multidisciplinary decision-making. “Relative contraindications” (e.g., tumors > 4 cm) implies that ablation may still be considered in imperative settings (e.g., solitary kidney) despite higher recurrence risks, whereas absolute contraindications preclude treatment.

**Table 2:** Comparison of minimally invasive ablative techniques for Renal Cell Carcinoma.

Feature	Radiofrequency Ablation (RFA)	Microwave Ablation (MWA)	Cryoablation (CA)	High-Intensity Focused Ultrasound (HIFU)
**Mechanism of Action**	High-frequency alternating current (400–460 kHz) inducing ionic oscillation (Resistive heating).	Electromagnetic waves (915 MHz or 2.45 GHz) causing water molecule oscillation (Dielectric heating).	Circulation of argon/nitrogen gases (Joule-Thomson effect) causing ice crystal formation.	Focused ultrasound waves (>1500 W/cm^2^) inducing thermal coagulative necrosis.
**Target Temperature**	50–90 °C	>60–100 °C (Rapid heating)	Low as −190 °C (Freezing)	>60 °C (Heat generation)
**Key Advantages**	Cauterizes vessels (minimizes bleeding) Heat-sink effect protects nearby structures Excellent long-term data for small tumors	Rapid and deeper heating Less influenced by heat-sink effect Shorter ablation times Effective in cystic tissue	Real-time visualization of “ice ball” Intrinsic analgesic effect (less pain) Precise control near collecting system	Completely non-invasive (no needle puncture) No risk of track seeding
**Key Limitations**	Heat-sink effect lowers efficacy near large vessels (≥3 mm) Efficacy relies on tissue impedance	Less predictable ablation shape Risk of thermal injury to adjacent organs due to high energy Limited real-time visualization	Risk of bleeding (no cauterization) Longer procedure time Rare risk of “cryoshock”	Experimental status Perinephric fat absorbs energy (poor efficacy in native kidneys) Respiratory motion issues
**Optimal Indication**	Small peripheral tumors (<3 cm) Tumors distant from vital structures	Larger tumors (3–4 cm) Cystic or vascularized lesions Perivascular tumors	Central/Hilar tumors Anterior masses Tumors near the ureter	Renal transplant recipients (absence of perinephric fat) Clinical trials only

Note: The “Key Advantages” and “Limitations” highlighted here represent the general consensus in current literature. The optimal indication depends on a combination of tumor size, location (central vs. peripheral), and proximity to critical structures (e.g., bowel, ureter). HIFU is currently distinct as the only non-invasive but experimental approach.

## Data Availability

No new data were created or analyzed in this study. Data sharing is not applicable to this article.

## Abbreviations

Abbreviations are used in this manuscript:

RCC	Renal Cell Carcinoma
RFA	Radiofrequency Ablation
MWA	Microwave Ablation
CA	Cryoablation
HIFU	High-Intensity Focused Ultrasound
CT	Computed Tomography
EAU	European Association of Urology
AUA	American Urological Association
DAMPs	Damage-Associated Molecular Patterns
