# Correction: Schubert et al. Minimally Invasive Ablation Strategies for Renal Cell Carcinoma Patients Ineligible for Surgery. *Life* 2026, *16*, 73

**DOI:** 10.3390/life16060919

**Published:** 2026-05-29

**Authors:** Or Schubert, Maria Chiara Sighinolfi, Filippo Gavi, Enrico Panio, Simone Assumma, Antonio Silvestri, Giuseppe Pallotta, Vincenzo Cavarra, Pierluigi Russo, Nazario Foschi, Eros Scarciglia, Alessandro Posa, Alessandro Maresca, Gaetano Gulino, Alessandro Cina, Chiara Ciccarese, Roberto Iacovelli, Luca Tagliaferri, Roberto Iezzi, Bernardo Rocco

**Affiliations:** 1 A. Gemelli University Hospital Foundation IRCCS, 00168 Rome, Italy; 2 Department of Urology, Fondazione Policlinico Universitario Agostino Gemelli IRCCS, 00168 Rome, Italy; 3 Department of Life Science, Health and Health Professions, Unilink University, 00165 Rome, Italy; 4 Department of Diagnostic Imaging and Radiation Oncology, Fondazione Policlinico Universitario Agostino Gemelli IRCCS, 00168 Rome, Italy; 5 Department of Oncology, Fondazione Policlinico Universitario Agostino Gemelli IRCCS, 00168 Rome, Italy

In the published article [1], Luca Tagliaferri was not included as an author. The corrected Author Contributions statement appears here. The authors state that the scientific conclusions are unaffected. This correction was approved by the Academic Editor. The original publication has also been updated.
